# Methicillin-Resistant *Staphylococcus aureus* T144: A Hypervirulent Model Strain for Infection Models

**DOI:** 10.3390/antibiotics14030270

**Published:** 2025-03-06

**Authors:** Changsi Mao, Yuan Liu, Meirong Song, Jianzhong Shen, Kui Zhu

**Affiliations:** 1 National Key Laboratory of Veterinary Public Health and Safety, College of Veterinary Medicine, China Agricultural University, Beijing 100193, China; maochangsi@cau.edu.cn (C.M.); meirong_song@cau.edu.cn (M.S.); sjz@cau.edu.cn (J.S.); 2 Jiangsu Co-Innovation Center for the Prevention and Control of Major Animal Infectious Diseases and Zoonoses, College of Veterinary Medicine, Yangzhou University, Yangzhou 225009, China; liuyuan2018@yzu.edu.cn

**Keywords:** MRSA T144, multidrug resistance, hypervirulence, animal models

## Abstract

**Background/Objectives**: Methicillin-resistant *Staphylococcus aureus* (MRSA) presents a major public health challenge due to its multidrug resistance and high virulence. Developing representative model strains is crucial for systematically assessing pathogenesis and antimicrobial therapies. **Methods**: The highly virulent MRSA strain T144, isolated from pigs, was characterized through whole-genome sequencing and antimicrobial susceptibility testing. Infection models were successfully established in *Galleria mellonella* and mice to evaluate virulence. A mouse lung infection model was specifically developed to assess bacterial load dynamics, immune responses, and the efficacy of vancomycin treatment. **Results**: MRSA T144 demonstrated broad-spectrum antibiotic resistance and high mortality rates in both *Galleria mellonella* and mouse models. Whole-genome sequencing identified multiple virulence-associated genes, including hemolysins and enterotoxins. The concentration of 7 × 10^8^ CFUs was optimized for establishing the mouse lung infection model. In the mouse lung infection model, MRSA T144 demonstrated rapid bacterial proliferation within the first 24 h, followed by a slower growth rate. Significant changes in immune markers were observed, with elevated levels of pro-inflammatory cytokines (IL-1β, IL-6, IL-8, IL-17a, TNF-α) and decreased IL-10 levels. Vancomycin treatment significantly improved survival rates and reduced bacterial load, confirming the model’s utility for antimicrobial efficacy studies. **Conclusions**: The successful establishment of MRSA T144 infection models provides a robust platform for investigating bacterial dynamics, immune responses, and antimicrobial efficacy against highly virulent MRSA strains. These findings highlight the potential of MRSA T144 as a valuable model for developing novel therapeutic strategies.

## 1. Introduction

*Staphylococcus aureus* is a significant opportunistic pathogen in both humans and animals, causing life-threatening infections such as necrotizing fasciitis, pneumonia, and sepsis [1,2,3,4]. Historically, *Staphylococcus aureus* was susceptible to a broad range of Gram-positive antibiotics, making it relatively manageable in clinical settings. However, the current landscape has altered dramatically due to the emergence of multidrug-resistant *Staphylococcus aureus*. The rise in methicillin-resistant *Staphylococcus aureus* (MRSA) has exacerbated the public health threat, as traditional antibiotics have become increasingly ineffective, leaving clinicians with limited therapeutic options [5,6,7]. MRSA has garnered global attention not only for its resistance to methicillin but for its broader resistance to antibiotics such as macrolides, tetracyclines, and chloramphenicol [8,9]. Globally, MRSA has caused the largest increase in deaths, directly causing 130,000 deaths in 2021, more than double the 57,200 deaths in 1990 [10,11]. Of particular concern is that MRSA has been reported to account for more deaths in the United States than AIDS [12,13], with highly pathogenic isolates representing a critical challenge.

To address these challenges and develop effective strategies against MRSA, a comprehensive understanding of its pathogenic mechanisms and an evaluation of novel antimicrobial agents are urgently needed [10]. The development of a MRSA infection model is critical for understanding the pathogen’s behavior and evaluating the efficacy of new antibacterial drugs. The MRSA infection model has played an important role in research, but it still faces some problems and challenges. Many animal models (such as the mouse peritonitis model, skin infection model, etc.) and in vitro models have been constructed for MRSA [14,15,16]. However, these models lack clinical relevance, and the strains, infectious doses, and infection routes used in different studies vary greatly, resulting in the limited comparability and reproducibility of model results. Notably, the acquisition of bacterial resistance is associated with a reduction in virulence due to the adaptive costs and compensatory evolution of drug-resistant bacteria [17,18]. This trade-off underscores the importance of identifying multidrug-resistant and highly pathogenic clinical isolates, which are essential for constructing reliable infection models and assessing the effectiveness of antimicrobial agents. However, few representative strains exhibit both high levels of drug resistance and pathogenicity, particularly among *Staphylococcus aureus* isolates [19,20].

In this study, the multidrug-resistant and highly pathogenic MRSA T144 strain was successfully isolated from the lungs of dead pigs with pneumonia. This case was acute, with symptoms including high fever (40–41 °C), shortness of breath, cough, and loss of appetite. Treatment with beta-lactam antibiotics and fluoroquinolones failed, and the pigs died. Anatomical examination found that the lungs of infected pigs showed obvious inflammation and consolidation, and the lung tissue was dark red. Due to its high virulence, MRSA T144 has been effectively employed to develop mouse infection models, including peritonitis–sepsis, thigh infection, and skin wound infection models. These models have been instrumental in evaluating the efficacy of antimicrobial agents, such as the biosurfactant-inspired heptapeptide Bacaucin-1 [21] and quaternary ammonium-capped gold nanoclusters (QA-AuNCs) [22] in our previous studies.

However, the drug susceptibility and pathogenic characteristics of MRSA T144 remain poorly understood, and standardized protocols for establishing pneumonia models using MRSA T144 have not yet been developed. Such standards are essential for the consistent and reliable evaluation of new antimicrobial agents against MRSA. This highlights the need for further comprehensive studies on this strain to facilitate the development of effective treatment strategies against this formidable pathogen.

## 2. Results

### 2.1. Analysis of Antibiotic Susceptibility and Pathogenicity

Antimicrobial susceptibility testing revealed that MRSA T144 exhibits resistance to nearly all commonly used antibiotics, including penicillin G, gentamicin, clindamycin, erythromycin, and tetracycline (Table 1). Encouragingly, MRSA T144 remains susceptible to linezolid, vancomycin, and rifampicin, which are critical drugs in the treatment of multidrug-resistant (MDR) Gram-positive bacterial infections. The minimum inhibitory concentrations (MICs) for these drugs fall well within the defined susceptible ranges, indicating their potential efficacy against this strain (Table 1). This finding suggests that these last-resort antibiotics could be effective in managing infections caused by MRSA T144. The resistance profile of MRSA T144 highlights the urgent need to explore alternative therapeutic options for MRSA infections. Simultaneously, the retained susceptibility to linezolid, vancomycin, and rifampicin provides a positive control for using this strain as a reliable model in antimicrobial efficacy studies.

**Figure 1:**
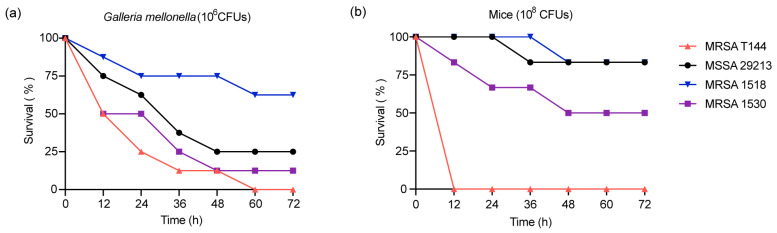
Evaluation of *S. aureus* virulence in *Galleria mellonella* (**a**) and mouse peritonitis–sepsis model (**b**). Survival curves were analyzed using the Mantel–Cox test. MRSA T144 demonstrated significantly higher mortality in mice compared to MSSA 29213 (*p* = 0.0006) and MRSA 1518/1530 (*p* = 0.0006 and 0.0022), respectively.

To investigate the pathogenicity of MRSA T144, an in vivo toxicity evaluation was conducted using *Galleria mellonella* larvae. For comparison, the methicillin-sensitive *Staphylococcus aureus* (MSSA) strain ATCC29213 and two previously reported animal-derived MRSA strains (1518 and 1530) were included as controls (Table S1). The virulence of the strains was assessed in the *Galleria mellonella* infection model [23]. *Galleria mellonella* larvae were infected with approximately 1 × 10^6^ colony-forming units (CFUs) of each strain, and survival rates were monitored over 72 h. At 24 h post-infection, only 25% of larvae infected with MRSA T144 survived, whereas the other three strains exhibited survival rates of 50% or higher. Remarkably, all MRSA T144-infected larvae died by 60 h post-infection. In contrast, at 72 h post-infection, the survival rates for MSSA 29213 and MRSA 1530 were 25% and 12.5%, respectively. The least lethal strain, MRSA 1518, showed a significantly higher survival rate of 62.5% (Figure 1a).

To further investigate the virulence of MRSA T144 in human-relevant mammalian infection models, an in vivo toxicity evaluation was performed using a mouse peritonitis–sepsis model. All mice were infected with the four selected strains, each at a dose of 1 × 10^8^ CFUs via intraperitoneal injection, and monitored for 72 h. Consistently, all mice infected with MRSA T144 died within 12 h. In contrast, the other three strains exhibited a lower mortality rate, with survival rates above 50%, particularly MSSA 29213 and MRSA 1518, which showed a mortality rate of only 12.5% (Figure 1b). Interestingly, the mortality rate caused by MSSA 29213 and MRSA 1530 in infected mice was lower than those observed in *Galleria mellonella*, which may be partly due to the higher susceptibility of larvae. Taken together, MRSA T144 demonstrates high pathogenicity and mortality rates in both *Galleria mellonella* and mice. To further characterize the virulence of MRSA T144, the pathogenicity of MRSA T144 was assessed by determining the LD_50_. The LD_50_ for MRSA T144 intranasally inoculated to mice was calculated to be 2.69 × 10^8^ CFUs, confirming its high virulence potential.

These findings not only confirm the significant drug resistance and strong pathogenicity of MRSA T144 but also establish a foundation for the development of new antimicrobial agents against MRSA infections. The high virulence, as demonstrated by the LD_50_ assay, underscores the urgent need for novel therapeutic strategies to combat such aggressive MRSA strains.

### 2.2. Antimicrobial Resistance Profiling and Phylogenetic Analysis of MRSA T144

Next, we performed the whole-genome sequencing of MRSA T144, generating a high-quality genome consisting of 2,881,741 base pairs with a G + C content of 32.95%. The chromosomal DNA sequences of MRSA T144 were deposited in NCBI GenBank with the accession number CP178536. Whole-genome sequencing revealed that MRSA T144 belongs to sequence type ST9 and carries SCCmec type XII. The analysis also showed that the strain harbors 26 drug resistance genes (Table S2), which confer resistance to various classes of antibiotics. These include the following: β-lactams (*mecA* and *blaZ*), aminoglycosides (*ant(4′)-Ia*, *ant(6′)-Ia*, *aac(6′)-Ie-aph(2″)-Ia*, *kdpD*), fluoroquinolones (*norA*, *norC*, *arlR*, *arlS*, *mgrA*, *sdrM*, and amino acid mutations in ParC S80F), tetracyclines (*mepR*, *tet*(45)), lincosamides (*lnuB*, *lsaE*), phenicols (*fexA*), diaminopyrimidines (*dfrG*), and phosphonic acid (*fosB*). Notably, the *lmrS* and *mgrA* genes encode components of a major facilitator superfamily (MFS)-type antibiotic efflux pump, while *ermC* encodes the 23S rRNA methyltransferase responsible for resistance to multiple antibiotics. Transposons Tn552 and Tn553 carry the *blaZ* gene, and transposon Tn558 carries the *fexA* gene. Collectively, these resistance genes and the associated phenotypic profile of MRSA T144 confirm that this isolate is a multidrug-resistant strain.

**Figure 2:**
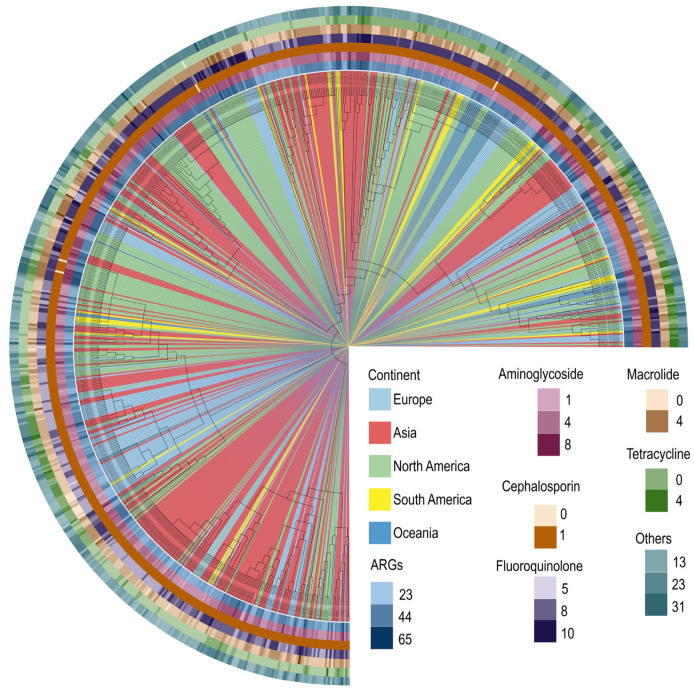
Phylogenetic tree of MRSA. A total of 120 single-copy genes were extracted from the genome sequences of 723 MRSA strains using the GTDB-TK tool. Phylogenetic analysis was conducted based on these genes. The classify step of GTDB-TK was used to determine the taxonomic classification of each genome and construct a phylogenetic tree reflecting the evolutionary relationships between the genomes. The tree was visualized using iTOL. The accession number of each strain is indicated in the tree.

Moreover, we obtained a total of 1381 *S. aureus* genomes from the NCBI RefSeq database, among which 722 MRSA genomes were selected based on the presence of the *mecA* gene (Table S4). The whole genomes were aligned, and a phylogenomic tree was constructed. We found that MRSA T144 shares higher homology with MRSA strains from Asia. MRSA strains with similar homology to MRSA T144 primarily originate from various provinces in China, including Beijing, Shanxi, Hubei, Guangzhou, Zhejiang, and Taiwan. Furthermore, these samples were derived from diverse sources, including human, animal, food, and environmental samples. To highlight the distribution of antibiotic resistance genes across strains, a heatmap displaying the number of resistance genes in each strain is shown at the periphery of the evolutionary tree (Figure 2).

### 2.3. Virulence Gene Characterization and Virulence Determinants of MRSA T144

**Figure 3:**
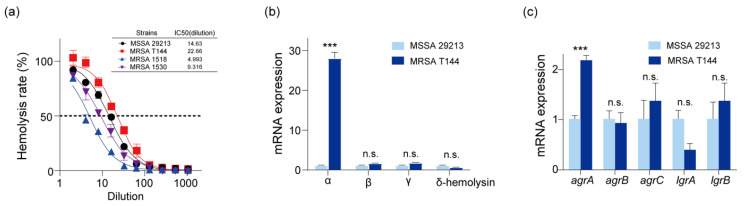
Hemolytic activity analysis of MRSA T144. (**a**) Hemolytic activity in RBC assay of four *S. aureus* strains. (**b**,**c**) The mRNA expression of four types of hemolysins and the related regulatory genes. Data are presented as means ± SD. Differences between two groups were determined by unpaired *t*-test and are denoted as follows: n.s.—not significant; *** *p* < 0.001.

Using the Virulence Finder 1.5 database (http://www.genomicepidemiology.org/, accessed on 10 April 2024) in conjunction with whole-genome sequencing analysis, we identified several virulence-associated genes in MRSA T144. These included metalloprotease (aureolysin)-encoding gene aur, hemolysin-encoding genes (*hla*, *hlb*, *hlg*, *hld*), Panton–Valentine leukocidin (PVL)-encoding genes (*lukS* and *lukF*), and multiple enterotoxin-encoding genes (*seu*, *sel*, *sem*, *seo*, *seg*, and *sen*). When our findings were compared with the virulence genes of 722 MRSA strains retrieved from the NCBI database, no significant differences were observed in the total number and types of virulence genes (Figure S1). Hemolysins have been recognized as critical factors in the pathogenicity of staphylococcal strains in other studies [24]. To further evaluate the hemolytic properties of MRSA T144, we compared its hemolytic activity with that of hemolysin-positive strains, including MSSA 29213 and two other MRSA strains. We found that MRSA T144 exhibited the highest hemolysis rate, with an IC50 dilution of 1:22.66, surpassing that of MSSA 29213 (Figure 3a).

To investigate the underlying cause of the strong hemolytic properties of MRSA T144, we first compared the mRNA expression levels of the four hemolysins in MRSA T144 with those in the model strain MSSA 29213. As shown in Figure 3b, the expression of the *α*-hemolysin gene increased nearly 30-fold in MRSA T144, while no significant changes were observed in the expression of *β*-, *γ*-, and *δ*-hemolysins. Consistently, we observed increased expression of the positive regulatory factor *agr*A and down-regulation of the negative regulatory factor *lrg*A for *α*-hemolysin (Figure 3c). In addition to mRNA gene expression analysis, we also sequenced the coding gene of α-hemolysin in both MSSA 29213 and MRSA T144. The sequencing analysis revealed a point mutation at position 608 (from A to T) in MRSA T144 compared to MSSA 29213 (Figure S2A). This point mutation is a nonsynonymous mutation that results in the amino acid substitution Gln203Leu. Homology modeling indicated that this point mutation occurs in a random coil of the transmembrane region, which may be associated with an enhanced membrane pore-forming activity of *α*-hemolysin (Figure S2B). Collectively, the increased expression and point mutation of *α*-hemolysin in MRSA T144 likely contribute to its hypervirulence both in vitro and in vivo.

### 2.4. Construction of Mouse Lung Infection Models

**Figure 4:**
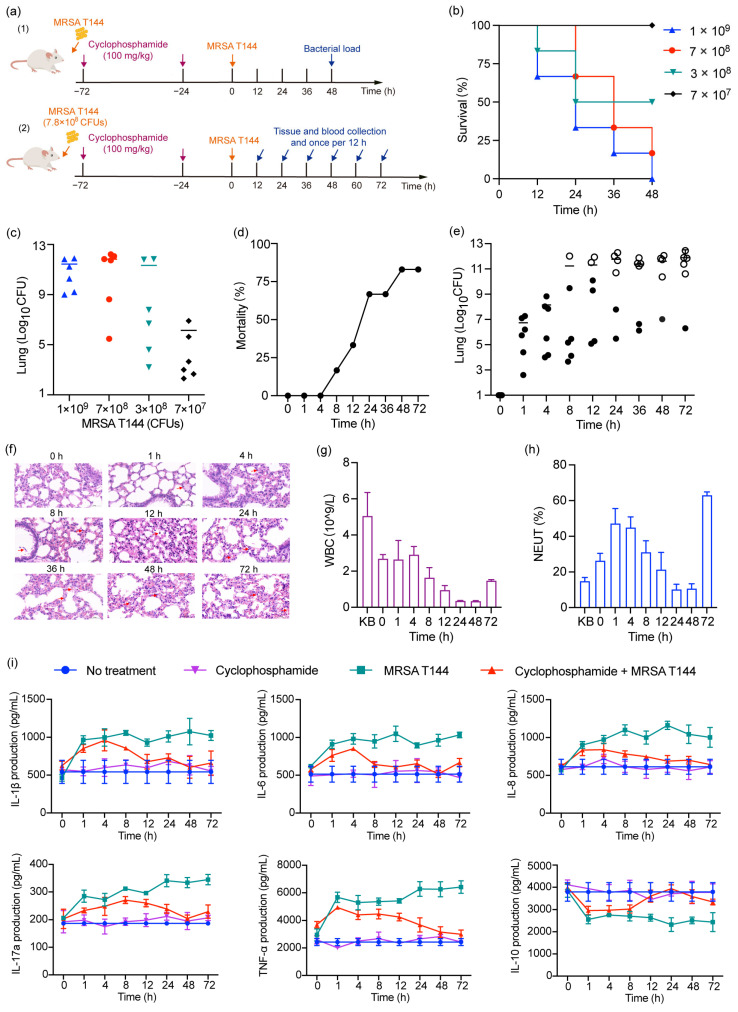
In vivo dynamic progression of MRSA T144. (**a**) The schematic diagram of intranasal infection with MRSA T144 in mice is divided into two parts: (1) Determination of the optimal bacterial inoculum by observing mortality within 48 h post-infection in mice inoculated with different concentrations of MRSA T144; (2) Investigation of the in vivo dynamic changes of MRSA T144 over a 72 h period following infection with 7.8 × 10^8^ CFUs. (**b**) Survival rate, and (**c**) lung bacterial load in mice at 48 h post-infection with different bacterial concentrations. (**d**) Survival rate, (**e**) lung bacterial load, (**f**) lung pathological changes at 40.0 × magnification (red arrows indicate inflammatory cell infiltration), (**g**) white blood cell count, (**h**) neutrophil count, and (**i**) concentrations of major inflammatory factors (IL-1β, IL-6, IL-8, IL-17a, TNF-α, and IL-10) were measured within 72 h after intranasal infection of mice with 7.8 × 10^8^ CFU of MRSA T144. Data are representative of six biological replicates; error bars represent SD.

To further assess the pathogenicity of MRSA T144 and the effectiveness of antibacterial agents against pneumonia, we established a mouse lung infection model (Figure 4a). Mice were intranasally inoculated with MRSA T144 at four different concentrations: 1 × 10^9^, 7 × 10^8^, 3 × 10^8^, and 7 × 10^7^ CFUs. Mice in the groups receiving 1 × 10^9^ and 7 × 10^8^ CFUs exhibited severe infections, with mortality rates of 100% and 83.3%, respectively (Figure 4b). However, the 1 × 10^9^ CFU concentration was deemed excessively high, resulting in poor health and elevated bacterial loads in the lungs (Figure 4c). Therefore, the 7 × 10^8^ CFU concentration was selected as the optimal inoculum for establishing a lethal lung infection model, providing a suitable foundation for subsequent evaluations of antimicrobial treatments. To investigate the dynamic progression of MRSA T144 within the lungs, we established a 72 h post-infection observation period. The mice were immunosuppressed via intraperitoneal administration of cyclophosphamide at a dose of 100 mg/kg, administered 24 and 72 h prior to infection. Six mice from each group were intranasally inoculated with 7.8 × 10^8^ CFUs of MRSA T144 in a 20 µL volume. Notably, the mice began to succumb at 8 h post-infection, with the highest mortality occurring between 8 and 24 h. The cumulative mortality rate reached 66.7% at 24 h and 83.3% at 72 h post-infection (Figure 4d). In terms of bacterial load, MRSA T144 proliferated rapidly within the lungs from 4 to 24 h post-infection. However, the bacterial growth rate slowed from 24 to 72 h post-infection (Figure 4e). Pathological examination revealed the progressive widening of the alveolar interstitium in infected mice over time. Alveolar spaces exhibited gradual atrophy with increased infiltration and accumulation of inflammatory cells and red blood cells (Figure 4f). At 72 h post-infection, the alveolar interstitium showed congestion, edema, and significant widening, accompanied by severe tissue hemorrhage. Moreover, the number of alveolar epithelial cells decreased, while the infiltration of red blood cells and inflammatory cells markedly increased. Furthermore, complete blood count (CBC) analyses indicated a significant decrease in white blood cells and neutrophils between 8 and 48 h (Figure 4g,h). By 72 h post-infection, both the total white blood cell count and the proportion of neutrophils began to recover.

To investigate the tissue preference of MRSA T144 when infecting mice, we performed an immunofluorescence assay. As shown in Figure S3, in the lung tissue of mice infected with the MRSA T144 strain, labeled with red fluorescence, the bacterial burden was higher than that of the uninfected group, predominantly in the alveolar septum and alveoli. The thickening of the alveolar septum was observed in the lungs of infected mice, with red fluorescence also present in the blood vessels within the septum. In the heart tissue, the red fluorescence signal was mainly distributed in the capillaries of the connective tissue between muscle fibers. In the kidneys, the red fluorescence signal was mainly localized to the glomerular blood vessels, with some also present in the renal tubules. The spleen of infected mice exhibited antigen stimulation, with an increase in the volume of splenic corpuscles, which was more pronounced compared to the uninfected group. The red fluorescence was mainly present in the spleen’s red pulp sinusoids. In the liver, red fluorescence was primarily distributed in the central vein and hepatic sinusoids. Notably, there was an absence of positive signals in the brain tissue, suggesting that the bacteria may not be able to cross the blood–brain barrier.

To investigate the impact of MRSA T144 infection on the immune response in mice, we assessed the temporal changes in key immune factors in a mouse pneumonia model. Each mouse was infected with 7.8 × 10^8^ CFUs of MRSA T144, and the concentrations of major immune factors in the serum and lungs were measured at 0, 1, 4, 8, 12, 24, 48, and 72 h post-infection. The results showed a time-dependent increase in the levels of pro-inflammatory cytokines, including IL-1β, IL-6, IL-8, IL-17a, and TNF-α (Figure 4i), which are associated with promoting the development of inflammation. IL-10, an anti-inflammatory cytokine, plays a crucial role in inhibiting the production of pro-inflammatory cytokines (such as TNF-α and IL-6), regulating the immune response, preventing the overactivation of the immune system, and maintaining immune tolerance. As expected, IL-10 levels showed a decreasing trend over time in response to the infection. Although the concentrations of these immune factors in the serum were lower than in the lungs, the trends in their temporal changes were consistent across both compartments. The establishment of the mouse lung infection model using MRSA T144 provides comprehensive insights into infection kinetics, bacterial growth patterns, and the host’s immune response. This model is crucial for a deeper understanding of MRSA T144 pathogenesis and will inform future strategies for the development of antimicrobial therapies.

### 2.5. Efficacy Evaluation of Vancomycin in the MRSA T144-Infected Mouse Model

**Figure 5:**
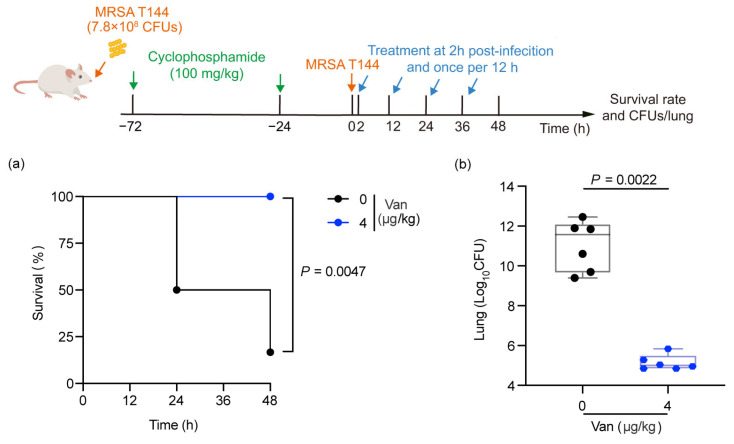
Efficacy of intranasal administration of vancomycin in MRSA T144-infected lungs (n = 6 per group). (**a**) Survival of mice infected with MRSA T144 following vancomycin treatment (*p* = 0.0047). (**b**) Reduction of bacterial load in the lungs by vancomycin in the mouse lung infection model (*p* = 0.0022).

To assess the potential of MRSA T144 as a model strain for evaluating the therapeutic efficacy of antimicrobial agents, we tested the efficacy of vancomycin, a drug to which MRSA T144 is sensitive in an infected mouse model. Following infection with MRSA T144 for 2 h, mice were treated with either PBS or 4 μg/kg vancomycin. The survival rate of the mice and the bacterial load in deceased mice were monitored over a 48 h period. The PBS-treated group was characterized by decreased mobility, curling up, and shortness of breath. The vancomycin-treated group gradually recovered mobility and became more active, and the respiratory rate gradually returned to normal. Compared to the PBS-treated group, the survival rate in the 4 μg/kg vancomycin-treated group was significantly higher (*p* = 0.0047, determined by the Mantel–Cox test). Correspondingly, the bacterial loads in all mice treated with 4 µg/kg vancomycin were significantly lower (*p* = 0.0022, determined by the Mann–Whitney test) (Figure 5).

## 3. Discussion

*Staphylococcus aureus*, particularly MRSA, has rapidly emerged as the most prevalent resistant pathogen, identified in numerous regions worldwide, including Europe, the United States, North Africa, the Middle East, and East Asia, representing a critical global health challenge [7,25]. In the European Union, MRSA causes around 150,000 hospital-acquired infections annually, leading to over 7000 deaths and incurring hospital costs of approximately EUR 380 million each year [26]. In Iran, according to available statistics, the positive rate of MRSA in *Staphylococcus aureus* is 43.0% [27]. Similarly, according to previous surveillance data, the prevalence of MRSA has ranged from 50% to 70% of all *Staphylococcus aureus* isolates in China [28]. These alarming trends underscore the urgent need to decipher MRSA’s pathogenic mechanisms and accelerate the development of novel therapeutics to curb its spread. In this study, we identified a multidrug-resistant (MDR), hypervirulent, livestock-associated, methicillin-resistant *S. aureus* (LA-MRSA) strain, designated T144, belonging to sequence type 9 (ST9). This strain exhibits strong toxicity toward both *Galleria mellonella* and mice. LA-MRSA has garnered significant attention due to its widespread presence in livestock and poultry, as well as its ability to colonize and infect individuals handling poultry, thereby increasing the risk of infection in these populations. For instance, the first case of human infection with pig-derived MRSA was reported in the Netherlands in 2005 [29]. Additionally, recent studies have shown that LA-MRSA clonal complex 398 (CC398) can be transmitted through various routes, including from the environment to humans, from livestock products to people, and from person to person, even in the absence of direct contact with livestock [30]. Numerous reports of LA-MRSA CC398 infections have since emerged globally [31,32,33]. Although human infections with pig-derived ST9 MRSA have been rare, the potential for transmission between animals and humans (such as between pigs and pig farmers) and within families (pig farmers and their families) should not be underestimated. Importantly, ST9 is the most prevalent LA-MRSA clonotype, with pigs serving as the primary host [34]. Notably, an isolate from a hospital in Guangzhou, China, was identified as ST9-t899 MRSA harboring SCCmec NT [35]. A surveillance study conducted in Taiwan identified nine human clinical MRSA strains of ST9, including seven SCC*mec* XII isolates and two SCC*mec* VT isolates [36,37]. These cases underscore the transmissibility of pig-source ST9 LA-MRSA and its stable colonization in humans. Furthermore, the phylogenetic analysis revealed that the T144 strain shares high homology with MRSA isolates from China, which have been sourced from humans, animals, food, and the environment. Given its multidrug resistance and hypervirulence, elucidating the high-pathogenicity mechanism of the ST9 LA-MRSA T144 strain and identifying effective antimicrobial agents to eliminate MRSA T144 strain infections will significantly reduce the potential threat of LA-MRSA to public health and healthcare systems.

Several community-associated methicillin-resistant *Staphylococcus aureus* (CA-MRSA) strains with enhanced virulence have been reported, such as USA300 [38,39] and JKD6159 [40]. However, relatively few hypervirulent LA-MRSA strains have been identified and studied, along with their critical virulence determinants. Two primary hypotheses have been proposed to explain the increased virulence in CA-MRSA strains: the acquisition of Panton–Valentine leukocidin (PVL) and the increased expression of core-genome-encoded toxins such as hemolysin. PVL is a member of the staphylococcal leukocidins and has been associated with skin infections. Since PVL genes (*lukS* and *lukF*) are typically absent from hospital-associated MRSA (HA-MRSA) strains and present in CA-MRSA, PVL was initially considered a potential contributor to CA-MRSA virulence [41]. However, animal studies have shown that CA-MRSA clones lacking PVL genes still exhibit significant virulence [42,43], raising doubts about the key role of PVL in CA-MRSA virulence. Similarly, the LA-MRSA T144 strain also contains the *lukS* and *lukF* genes, which are 100% identical to those found in CA-MRSA strains, yet the potential contribution of these genes to virulence remains unclear. In this study, we demonstrated that the hypervirulence of MRSA T144 is strongly associated with increased expression and a nonsynonymous mutation in the α-hemolysin gene. α-Hemolysin is a self-assembling, channel-forming toxin produced by *S. aureus*. This finding is consistent with observations that the hyperexpression of α-hemolysin contributes to the enhanced virulence of ST93 CA-MRSA. α-Hemolysin is typically secreted as an inactive 33.2 kDa soluble monomer, which subsequently self-assembles into a 232.4 kDa heptamer capable of inducing host cell lysis and death [44,45]. In addition, α-hemolysin has been shown to promote the proliferation, spread, and lethality of Gram-negative bacteria by preventing the acidification of macrophage phagosomes, thereby facilitating co-infections in mixed-pathogen lung infection models [46]. Therefore, MRSA isolates with elevated α-hemolysin expression warrant urgent attention due to their potential to exacerbate infections.

Importantly, the hypervirulent LA-MRSA strain MRSA T144 offers a promising model for screening potential advanced antimicrobial agents. The pathogenicity, infection kinetics, and host immune responses of MRSA T144 were thoroughly investigated by establishing a lung infection model using this strain. We observed high mortality resulting from high-concentration MRSA T144 inoculation, consistent with previous studies. Furthermore, we refined the infectious dose and determined that 7.5 × 10^8^ CFUs is the optimal inoculation amount for establishing a fatal lung infection model; the mice began to succumb at 8 h post-infection, with the highest mortality occurring between 8 and 24 h, and the cumulative mortality rate reached 66.7% at 24 h. In terms of bacterial load, MRSA T144 proliferated rapidly within the lungs from 4 to 24 h post-infection. However, the bacterial growth rate slowed from 24 to 72 h post-infection, providing an accurate dose reference for future studies. In terms of immune response, we found that the levels of pro-inflammatory factors increased following MRSA infection, while the level of the anti-inflammatory factor IL-10 decreased. Studies have shown that MRSA carrying PVL can up-regulate the expression of pro-inflammatory cytokines. A reduction in IL-10 levels promotes an IFN-γ-dominated cytokine response, leading to mortality in animals with acute pneumonia [47,48]. This provides a solid foundation for constructing a mouse infection model. We also confirmed the distribution of MRSA T144 in various mouse tissues and observed bacterial aggregation in the alveoli and blood vessels. Additionally, we evaluated the ability of MRSA T144 to breach the blood–air barrier and enter the bloodstream, potentially reaching other organs. Our findings suggest that this strain may not be able to cross the blood–brain barrier. Finally, in evaluating the efficacy of vancomycin, we demonstrated that vancomycin significantly improved the survival rate of MRSA-infected mice. This study further validates the efficacy of vancomycin and provides experimental support for its clinical use in treating MRSA infections.

These results demonstrate the significant potential of hypervirulent MRSA T144 in a pneumonia infection model. Importantly, we hope that novel antimicrobial agents can be identified through the use of MRSA T144-associated infection models, which could help mitigate the potential threat of LA-MRSA in both humans and animals. However, further mechanistic studies investigating the virulence determinants of MRSA T144 are highly encouraged.

## 4. Materials and Methods

### 4.1. Animals

*Galleria mellonella* larvae, each weighing approximately 300 mg, were purchased from Tianjin Huiyude Biotech Company (Tianjin, China) and maintained on woodchips in the dark at 15 °C until use. They were acclimatized for at least one week before being used in experiments to ensure that they were in a stable physiological state. Six- to eight-week-old female BALB/c mice (18–20 g) maintained in specific pathogen-free (SPF) conditions were obtained from SPF (Beijing) Biotechnology Co., Ltd.(Beijing, China). Mice were housed in standard polycarbonate cages with wood shavings as bedding material. The cages were placed in a controlled environment with a 12 h light/dark cycle, a temperature of 22 ± 2 °C, and a relative humidity of 50–60%, maintained in specific pathogen-free (SPF) conditions. Mice were acclimated to standardized environmental conditions for one week prior to infection. All animal study protocols were conducted in accordance with the relevant guidelines and regulations (ID: SKLAB-B-2010-003). The laboratory animal use license number is SYXK-2021-0012, certified by the Beijing Association for Science and Technology. The experimental protocols were approved by the Laboratory Animal Ethics Committee of China Agricultural University (AW03214202-2-02).

### 4.2. Antimicrobial Susceptibility Test

The antimicrobial susceptibility of representative antimicrobial agents against MRSA T144 was determined using the broth microdilution method or the agar dilution method, following the CLSI 2024 guidelines [49]. Briefly, antimicrobial agents were serially diluted 2-fold in cation-adjusted Mueller–Hinton Broth (MHB, Beijing Land Bridge) and mixed with an equal volume of bacterial suspensions in MHB, containing approximately 1.5 × 10⁶ colony-forming units (CFUs)/mL, in a clear UV-sterilized 96-well microtiter plate (Corning). Additionally, disks containing appropriate concentrations of antimicrobial agents were placed on Mueller–Hinton Agar (MHA) plates inoculated with bacterial suspensions. The MHB medium alone and MHB medium containing MRSA T144 were used as negative and positive controls, respectively. The standard quality control strain, *S. aureus* ATCC 29213, was used according to the CLSI 2024 guidelines. The antimicrobial agents were tested at a concentration of 0.03–128 μg/mL for a total of 13 concentration gradients. After 18 h of incubation at 37 °C, the minimum inhibitory concentrations (MICs) and inhibition zone diameters were recorded.

### 4.3. Virulence Evaluation in Galleria Mellonella and Mice

The virulence of MSSA 29213, MRSA T144, MRSA 1518, and MRSA 1530 was first assessed using the *Galleria mellonella* infection model. *Galleria mellonella* larvae were randomly divided into four groups (n = 8 per group) and infected with 10 µL of *S. aureus* suspension (1.0 × 10^6^ CFUs) at the right posterior gastropod. The infected larvae were then maintained at 37 °C in a humidified incubator. Survival rates were monitored every 12 h for a total of 72 h. The survival rates of the larvae were recorded over a 72 h period. For the mouse peritonitis–sepsis model, female BALB/c mice (n = 6 per group) were infected with 1.0 × 10^8^ CFUs of *S. aureus* suspension via intraperitoneal injection. Mice were observed every 12 h after infection. Health status was assessed based on activity level, respiratory rate, and appetite. Mortality was recorded as the primary endpoint. The survival rates of infected mice were monitored for 72 h. Mice were euthanized using a gradual-fill CO_2_ chamber. We followed the guidelines of the China Association for Laboratory Animal Science. CO_2_ was pre-administered into a transparent chamber. After 5 min, the mice were then placed inside the chamber. Subsequently, CO_2_ was introduced into the chamber for another 5 min. Once the cessation of movement and breathing in the mice was observed, the gas supply was stopped. The mice were monitored for an additional 2 min to confirm death.

### 4.4. LD_50_ Determination

After a 3-day acclimatization period, 50 BALB/c mice (equally divided between males and females) were randomly assigned to 5 groups, with 10 mice per group. The MRSA T144 strain was inoculated into Brain Heart Infusion (BHI) broth and cultured overnight at 37 °C. Following incubation, the bacterial culture was centrifuged at 6000 rpm for 10 min, and the bacterial pellet was washed three times with PBS. The bacteria were then resuspended in 1 mL of PBS, and CFUs were enumerated. Based on the CFU count, the bacterial suspension was serially diluted in a 1:10 gradient, resulting in dilution factors of 10^0^, 10^1^, 10^2^, 10^3^, and 10^4^. Mice in the experimental groups were intranasally inoculated with bacterial suspensions of varying concentrations, while the control group was treated with PBS. Seven days post-inoculation, the survival and mortality rates of the mice were assessed. The LD50 value was determined using the modified Reed–Muench method, calculated as follows:

LD_50_ = log^−1^[Xm − i(ΣP − 0.5)].

Xm represents the logarithmic value of the dose in the highest-dose group.
i is the logarithmic ratio of the doses between adjacent high- and low-dose groups.
ΣP is the cumulative sum of the mortality rates across all groups.

### 4.5. Whole-Genome Sequencing

Genomic DNA was extracted using the SDS method [50]. The extracted DNA was assessed via agarose gel electrophoresis and quantified using a Qubit^®^ 2.0 Fluorometer (Thermo Scientific, Waltham, MA, USA). A total of 1 μg of DNA per sample was used as input material for the DNA sample preparation. Sequencing libraries were generated using the NEBNext^®^ Ultra™ DNA Library Prep Kit for Illumina (New England Biolabs (NEB), Ipswich, MA, USA) following the manufacturer’s protocol. Indexing was performed to attribute sequences to individual samples. Briefly, the DNA sample was fragmented by sonication to a size of 350 bp. The DNA fragments were then end-polished, A-tailed, and ligated with the full-length adaptors for Illumina sequencing, followed by PCR amplification. Finally, the PCR products were purified using the AMPure XP system, and the libraries were analyzed for size distribution using the Agilent2100 Bioanalyzer and quantified via real-time PCR. The raw data obtained by sequencing were filtered. Open source software fastp (https://github.com/OpenGene/fastp, accessed on 15 March 2024) was used for data quality control. After preprocessing, clean data were obtained, which were assembled by SOAP denovo (version 2.04), SPAdes, and ABySS assembly software, and finally integrated by CISA software 3.0. Gapclose (Version: 1.12) was used to optimize the preliminary assembly results and fill holes.

The whole genome of MRSA T144 was sequenced using the Illumina NovaSeq PE150 platform at Beijing Novogene Bioinformatics Technology Co., Ltd.(Beijing, China). Following this, the detection of SCCmec in MRSA T144 was performed using the Center for Genomic Epidemiology (http://www.genomicepidemiology.org, accessed on 10 April 2024).

### 4.6. Bioinformatics

Gene function annotation was performed using RAST (https://rast.nmpdr.org, accessed on 10 April 2024). Antimicrobial resistance genes were identified through the Comprehensive Antibiotic Resistance Database (https://card.mcmaster.ca, accessed on 10 April 2024) and ResFinder 4.1 (https://cge.food.dtu.dk/services/ResFinder/, accessed on 10 April 2024). Virulence genes were identified using the Virulence Factor Database (VFDB). Transposons were identified using The Transposon Registry (https://transposon.lstmed.ac.uk, accessed on 10 April 2024).

To screen for methicillin-resistant *Staphylococcus aureus*, we obtained the genomes of all *S. aureus* strains from the NCBI RefSeq database. Gene coding regions and protein sequences for these genomes were predicted using Prokka software (version v1.14.5). A local protein sequence database was constructed with Diamond software (version 5). The protein sequence of the *mec*A gene was downloaded from the NCBI non-redundant database and used as a query sequence for Diamond blastp alignment against the local database. Finally, based on the mecA gene, we identified 722 MRSA genomes.

We extracted 120 single-copy genes from the genomic sequences using the GTDB-TK tool. Multi-sequence alignment of these genes was performed to identify and compare the location and sequence of each gene across different genomes. The classify step of GTDB-TK was utilized to determine the taxonomic classification of each genome, and a phylogenetic tree was constructed to reflect the evolutionary relationships among the genomes.

We used the local version of annotation software (RGI 6.0.3) provided by the CARD database to identify drug resistance genes in the obtained protein sequences. In addition, we utilized Diamond software to create a local database of VFDB protein sequences and employed the Diamond blastp command to annotate the protein sequence files for virulence genes.

To predict the three-dimensional structure of α-hemolysin, the amino acid sequence of the protein was obtained and submitted to SWISS-MODEL for homology modeling. Hemolysin of the *Staphylococcus aureus* Mu50 strain (pdb number:3ANZ) was used as template. The resulting model was then optimized and visualized using PyMOL 2.6.

### 4.7. Hemolysis Assay

*S. aureus* was cultured in 1 mL of BHI broth for 8 h and then centrifuged at 13,000× *g* for 5 min. The bacterial pellet was resuspended in fresh medium for the hemolysis assay. The hemolytic activity of the bacterial suspension was determined according to a previously reported method [51]. Sheep red blood cells were prepared from fresh, sterile defibrinated sheep blood (Land Bridge Technology, Beijing, China) and then diluted to 2% in Phosphate-buffered saline (PBS, 0.01 M, pH 7.4), and equal volumes of bacterial suspension (10^8^ CFU/mL) with various dilutions (1–1000) were incubated at 37 °C for 1 h. PBS, with or without 0.2% Triton X-100, served as the negative and positive controls, respectively. The absorbance of the released hemoglobin was measured at 576 nm using a multifunctional microplate reader (Spectramax M5, Molecular Devices). The hemolysis rate was calculated using the following formula:

Hemolysis (%) = [(OD_576 sample_ − OD_576 blank_)/(OD_576 0.2% Triton X-100_ − OD_576 blank_)] × 100%

### 4.8. RT-PCR Analysis

*S. aureus* ATCC 29213 and MRSA T144 were cultured overnight in LB broth and then diluted 1:1000 into 1 mL of fresh LB. After the bacterial cells reached the mid-log phase (OD_600_ = 0.5) at 37 °C, total RNA was extracted using the EASYspin Plus kit (Aidlab, Biotechnology Co., Ltd., Beijing, China) and quantified by measuring the absorbance ratio at 260 nm/280 nm using a Nanodrop spectrophotometer (Thermo Scientific). Prior to cDNA synthesis, RNA concentrations from all bacterial samples were normalized to the same concentration. Reverse transcription of 1 μg of extracted RNA was performed using the PrimeScript™ RT Reagent Kit (Takara Bio (Beijing) Co., Ltd., Beijing, China), following the manufacturer’s instructions.

The mRNA levels of α-, β-, γ-, and δ-hemolysin (*hla*, *hlb*, *hlg*, *hld*), *agr*A/B/C, and *lrg*A/B, relative to the control genes in *S. aureus*, were quantified by RT-PCR. RT-PCR was performed using the SYBR Green qPCR Kit (Takara Bio (Beijing) Co., Ltd., Beijing, China), with primers designed as listed in Table S3. Thermal cycling was conducted using a two-step PCR amplification procedure: initial denaturation at 95 °C for 30 s, followed by 40 cycles of 95 °C for 5 s and 60 °C for 34 s. RT-PCR was carried out using the ABI QuantStudio™ 7 detection system (Applied Biosystems, Foster City, CA, USA). Gene expression fold changes were calculated using the 2^−ΔΔCt^ method.

### 4.9. Mouse Lung Infection Model

To establish a lethal lung infection model with MRSA T144, mice were immunosuppressed via intraperitoneal injection of cyclophosphamide at a dose of 100 mg/kg, administered 24 and 72 h prior to infection. Subsequently, the mice were randomly assigned to four groups, with six animals per group. Each group was infected with MRSA T144 at concentrations of 1 × 10^9^, 7 × 10^8^, 3 × 10^8^, and 7 × 10^7^ CFUs, respectively. Under anesthesia (each mouse was anesthetized with a 3.5% isoflurane gas mixture at a gas flow rate of 3 L/min for 3 min), 20 µL of the respective bacterial suspension was slowly administered into the same nostril of each mouse to ensure consistent delivery. Survival rates were monitored for 48 h post-infection. At the end of the observation period, mice were euthanized, and their lungs were harvested to quantify the bacterial load in the organs. This assessment aimed to determine the optimal inoculum dose for establishing a reliable MRSA T144 lung infection model.

### 4.10. Dynamic Analysis of MRSA T144 in Mouse Lungs

The treatment of the animals before the experiment was the same as that in the mouse lung infection model. Subsequently, each group of six mice was intranasally inoculated with MRSA T144 at a concentration of 7.8 × 10^8^ CFUs in a 20 µL volume per mouse. Mortality rates were meticulously recorded at nine distinct time points: 0, 1, 4, 8, 12, 24, 36, 48, and 72 h post-infection. At each designated time point, surviving mice were euthanized humanely, and their lungs were harvested for bacterial load quantification using colony-forming unit assays. Additionally, lung tissues were subjected to comprehensive histopathological examinations to assess tissue damage and inflammatory responses. Blood samples were also collected for complete blood count (CBC) analyses to evaluate systemic hematological changes induced by the infection.

### 4.11. Immunofluorescence

Tissues (heart, liver, spleen, kidney, lung, and brain) harvested from euthanized mice were embedded in optimal cutting temperature (OCT) compound (Tissue-Tek^®^, Sakura Finetek USA, Torrance, CA, USA), flash-frozen, and sectioned into 10 μm slices using a cryostat (Leica Biosystems, (Shanghai) Co., Ltd., Shanghai, China). Sections were fixed in acetone at 4 °C for 10 min, subjected to citrate-based antigen retrieval via microwave heating at 95–100 °C for 5 min, and treated with 3% H_2_O_2_ to quench endogenous peroxidase. After blocking with goat serum at 37 °C for 30 min, slices were incubated overnight at 4 °C with a rabbit anti-*Staphylococcus aureus* SPA primary antibody (1:800; Cat. No. ab20920, Abcam, Cambridge, UK), followed by Alexa Fluor 594-conjugated secondary antibody (1:500; Invitrogen, A-11012) at 37 °C for 30 min. Nuclei were stained with DAPI (1 μg/mL; Sigma-Aldrich, (Merck KGaA), St. Louis, MO, USA) for 5 min at RT, and mounted with ProLong™ Diamond Antifade Mountant (Invitrogen, Carlsbad, CA, USA). Fluorescence signals were visualized using an Olympus fluorescence microscope (Olympus Tokyo, Japan). Image analysis was performed using ImageJ2 software (Wayne Rasband, National Institute of Mental Health, National Institute of Health, Bethesda, MD, USA) to merge the images.

### 4.12. Determination of Inflammatory Factors in MRSA T144 Infection

Mice were divided into four groups, including the no treatment group, the cyclophosphamide-only group, the MRSA T144 infection group, and the cyclophosphamide plus MRSA T144 infection group. Each group included three mice per time point, with a total of nine time points. Mice in the cyclophosphamide group and the cyclophosphamide plus MRSA T144 infection group received an intraperitoneal injection of cyclophosphamide (100 mg/kg) 24 h and 72 h prior to infection with MRSA, respectively. Mice in the MRSA T144 infection group and the cyclophosphamide plus MRSA T144 infection group were infected via nasal drip with 7.8 × 10^8^ CFUs per mouse. Serum and lung tissues were collected at 0, 1, 4, 8, 12, 24, 48, and 72 h after infection. Orbital venous blood collection was centrifuged at 4000× *g* for 30 min, and the supernatant was collected and used to determine the concentrations of inflammatory factors in the serum. Meanwhile, lung tissues were ground in liquid nitrogen to prepare a homogenate, adjusted to a certain concentration, for the detection of the concentrations of inflammatory factors in the lung. The concentrations of IL-1β, IL-6, IL-8, IL-10, IL-17a, and TNF-α were analyzed using ELISA kits (Nanjing Jiancheng Bioengineering Institute, Nanjing, China).

### 4.13. Rescue of Infected Mice with Vancomycin

Female BALB/c mice (n = 6 per group, 18–20 g) were intranasally inoculated with MRSA T144 at a dose of 7.8 × 10^8^ CFUs per mouse in a 20 µL volume. Two hours post-infection, mice received an intranasal dose of vancomycin (4 µg/kg) in a 20 µL volume per mouse. A second dose was administered 12 h post-infection, followed by additional doses every 12 h, for a total of four doses. During the treatment period, survival rates were recorded, and bacterial loads in deceased mice were quantified. At 48 h post-infection, surviving mice were humanely euthanized, and their lungs were collected to determine the bacterial load.

### 4.14. Statistical Analyses

Statistical analyses were performed using GraphPad Prism 10 and SPSS 27.0.1 software. Data are presented as means ± standard deviation (SD). Survival curves were compared using the Mantel–Cox test. For other comparisons, unpaired *t*-tests were used for two groups, and one-way ANOVA was applied for multiple groups. *p*-values were considered significant at * *p* < 0.05, ** *p* < 0.01, and *** *p* < 0.001.

## 5. Conclusions

We isolated the broad-spectrum antibiotic-resistant MRSA strain T144 from a pig. Whole-genome sequencing identified several drug resistance genes and virulence-related genes, among which point mutations in the α-hemolysin gene enhanced hemolytic activity. The utility of MRSA T144 as a reliable model for antimicrobial efficacy studies in vitro and in vivo suggests that novel therapeutic strategies against this highly virulent and broad-spectrum-resistant MRSA strain are urgently needed.

## Figures and Tables

**Table 1:** Antibiotic resistance characteristics of fatal MRSA T144.

Antibiotics	MIC (µg/mL)	Antibiotics	Inhibition Zone Diameter (mm)
Amoxicillin	16	Penicillin G (10 units)	6
Ceftiofur	8	Quinupristin/dalfopristin	17
Cefoxitin	8	Tetracycline (30 µg)	8
Clindamycin	>128	Fusidic acid (10 µg)	25
Doxycycline	16	Gentamicin (10 µg)	12
Enrofloxacin	2	Ciprofloxacin (5 µg)	16
Erythromycin	>128	Rifampicin (5 µg)	29
Chloramphenicol	32		
Florfenicol	64		
Linezolid	1		
Vancomycin	0.5		

## Data Availability

The original contributions presented in this study are included in the article/Supplementary Materials. Further inquiries can be directed to the corresponding authors.

## Supplementary Materials

The following supporting information can be downloaded at https://www.mdpi.com/article/10.3390/antibiotics14030270/s1, Figure S1: Number and species of virulence genes in MRSA; Figure S2: Nonsynonymous SNP in the α-hemolysin encoding gene of MRSA T144, relative to MSSA ATCC29213; Figure S3: Distribution characteristics of bacteria in organs of mouse model; Table S1: Characteristics of S. aureus iso-lates used in this study; Table S2: Resistance genes identified in MRSA T144; Table S3: Optimized primers used for RT-PCR analysis; Table S4: Strain number and Location of 723 strains in phylo-genetic tree.

## Abbreviations

The following abbreviations are used in this manuscript:

MRSA	methicillin-resistant *Staphylococcus aureus*
VRSA	vancomycin-resistant *Staphylococcus aureus*
QA-AuNCs	quaternary ammonium-capped gold nanoclusters
MDR	multidrug-resistant
MICs	minimum inhibitory concentrations
MSSA	methicillin-sensitive *Staphylococcus aureus*
PVL	Panton–Valentine leukocidin
RBCs	red blood cells
CBC	complete blood count
LA-MRSA	livestock-associated methicillin-resistant *Staphylococcus aureus*
CA-MRSA	community-associated methicillin-resistant *Staphylococcus aureus*
HA-MRSA	hospital-associated MRSA
SPF	specific pathogen-free
MHB	Mueller–Hinton Broth
MHA	Mueller–Hinton Agar
BHI	Brain Heart Infusion
VFDB	Virulence Factor Database
