# Supplementary material for: Methicillin-Resistant Staphylococcus aureus T144: A Hypervirulent Model Strain for Infection Models
Source: Antibiotics (Basel). 2025 Mar 6;14(3):270. doi: 10.3390/antibiotics14030270 (PMC11939158; doi:10.3390/antibiotics14030270)
Supplement: Supplementary file 1 [file antibiotics-14-00270-s001.zip › antibiotics-3484393-supplementary.pdf]

Supporting Information for

Methicillin-Resistant *Staphylococcus aureus* T144: A Hypervirulent Model Strain for Infection Models

Changsi Mao[1], Yuan Liu[2], Meirong Song[1], Jianzhong Shen[1,*] and Kui Zhu[1,*]

1 National Key Laboratory of Veterinary Public Health and Safety, College of Veterinary Medicine, China Agricultural University, No. 2 Yuanmingyuan West Road, Beijing 100193, China.; maochangsi@cau.edu.cn
2 Jiangsu Co-Innovation Center for the Prevention and Control of Major Animal Infectious Diseases and Zoonoses, Institute of Comparative Medicine, College of Veterinary Medicine, Yangzhou University, Yangzhou 225009, Jiangsu, China.; liuyuan2018@yzu.edu.cn
* Correspondence: Prof. Jianzhong Shen and Prof. Kui Zhu. sjz@cau.edu.cn; zhuk@cau.edu.cn.

This PDF file includes:

Tables S1 to S3

Figures S1 to S3

Supplementary Table S1 Characteristics of *S. aureus* isolates used in this study.

Isolates	Origin	Isolation site	PFGE type[b]	MLST type[c]
S. aureus 29213	ATCC[a]	-	-	-
MRSA T144	Ningxia	Nose	F	9
MRSA 1518	Guangdong	Lung	C2	9
MRSA 1530	Guangdong	Lung	C3	63

[a]ATCC, American Type Culture Collection.

[b]PFGE type was determined by *SmaI* pulsed-field gel electrophoresis (PFGE).

[c]Multilocus sequence typing (MLST) types were predicted based on the MLST web server (http://saureus.mlst.net).

Supplementary Table S2 Resistance genes identified in MRSA T144.

Gene	Best Identities(%)	Drug Class	Resistance Mechanism
mecA	99.7	penicillin beta-lactam	antibiotic target replacement
norC	99.57	fluoroquinolone antibiotic; disinfecting agents and antiseptics	antibiotic efflux
dfrG	100	diaminopyrimidine antibiotic	antibiotic target replacement

fosB	99.28	phosphonic acid antibiotic	antibiotic inactivation
norC	70.64	fluoroquinolone antibiotic; disinfecting agents and antiseptics	antibiotic efflux
arlR	100	fluoroquinolone antibiotic; disinfecting agents and antiseptics	antibiotic efflux
arlS	100	fluoroquinolone antibiotic; disinfecting agents and antiseptics	antibiotic efflux
norA	100	fluoroquinolone antibiotic	antibiotic efflux
mgrA	100	fluoroquinolone antibiotic; cephalosporin; penicillin beta-lactam; tetracycline antibiotic; peptide antibiotic; disinfecting agents and antiseptics	antibiotic efflux
mepR	100	glycylcycline; tetracycline antibiotic	antibiotic efflux
ant(4')-Ia	99.6	aminoglycoside antibiotic	antibiotic inactivation

lnuB	99.63	lincosamide antibiotic	antibiotic inactivation
lsaE	100	lincosamide antibiotic; streptogramin antibiotic; pleuromutilin antibiotic	antibiotic target protection
ant(6)-Ia	100	aminoglycoside antibiotic	antibiotic inactivation
tet(45)	77.14	tetracycline antibiotic	antibiotic efflux
ermC	99.59	macrolide antibiotic; lincosamide antibiotic; streptogramin antibiotic; streptogramin A antibiotic; streptogramin B antibiotic	antibiotic target alteration
aac(6')-Ie-aph(2'')-Ia	100	aminoglycoside antibiotic	antibiotic inactivation
PC1	100	penicillin beta-lactam	antibiotic inactivation
sdrM	100	fluoroquinolone antibiotic; disinfecting agents and antiseptics	antibiotic efflux

sepA	96.82	disinfecting agents and antiseptics	antibiotic efflux
lmrS	99.38	macrolide antibiotic; aminoglycoside antibiotic; oxazolidinone antibiotic; diaminopyrimidine antibiotic; phenicol antibiotic	antibiotic efflux
kdpD	98.76	aminoglycoside antibiotic	antibiotic efflux
vanT	33.88	glycopeptide antibiotic	antibiotic target alteration
blaZ	95.02	penam	antibiotic inactivation
fexA	99.37	phenicol antibiotic	antibiotic efflux

Supplementary Table S3 Optimized primers used for RT-PCR analysis.

Genes	Sequence (5′→3′)
α-hemolysin (*hla*)	CTTGGAACCCGGTATATGGC
	CGAAGTCTGGTGAAAACCCTG
β-hemolysin (*hlb*)	AATCGCTACGCCACCATCTT
	CACCTGTACTCGGTCGTTCT
γ-hemolysin (*hlg*)	AGCCCCTTTAGCCAATCCAT
	TGATTTCTGCACCTTGACCGA
δ-hemolysin (*hld*)	TATGGGGAACGACACTTTGGG
	ATACCTACCCAGCCCATCACT
agrA	CCAACTGGGTCATGCTTACG
	GCCCTCGCAACTGATAATCCT
agrB	TGCATCCCTAAACGTACTTGC
	TGACCAGTTTGCCACGTATCT
agrC	CCTAAACCACGACCTTCACCT
	CCCTATCATTCGCGTTGCAT

lrgA	GACGCATCAAAACCAGCACA
	GATGCAGGCATAGGAATTGGC
lrgB	TTGCCCGAGGATTAGCACTT
	CAGGCACAACTGCTACAACAA
16sRNA	CCGTGGGCTTTCACATCAGA
	TAGGTGGCAAGCGTTATCCG

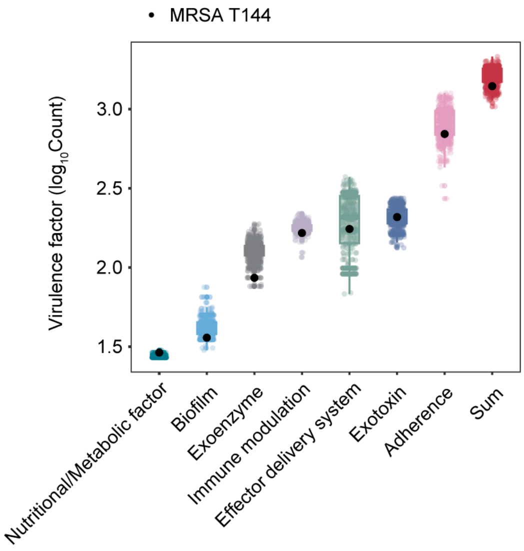

Supplementary Figure S1 Number and species of virulence genes in MRSA. A local library of VFDB protein sequences was established using Diamond software, and 723 protein sequence files were annotated by Diamond blastp command.

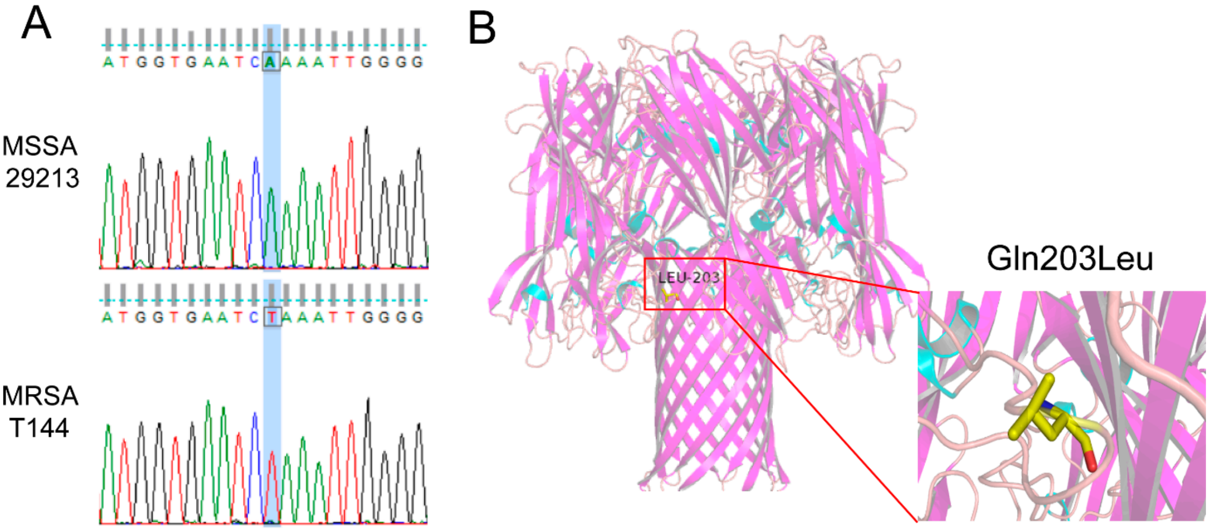

Supplementary Figure S2 Nonsynonymous SNP in the α-hemolysin encoding gene of MRSA T144, relative to MSSA ATCC29213. (A) Sequence determination. (B) Homology model of α-hemolysin. The 3D structure of α-hemolysin was predicted using SWISS-MODEL, and the structural figure was refined and generated using PyMOL.

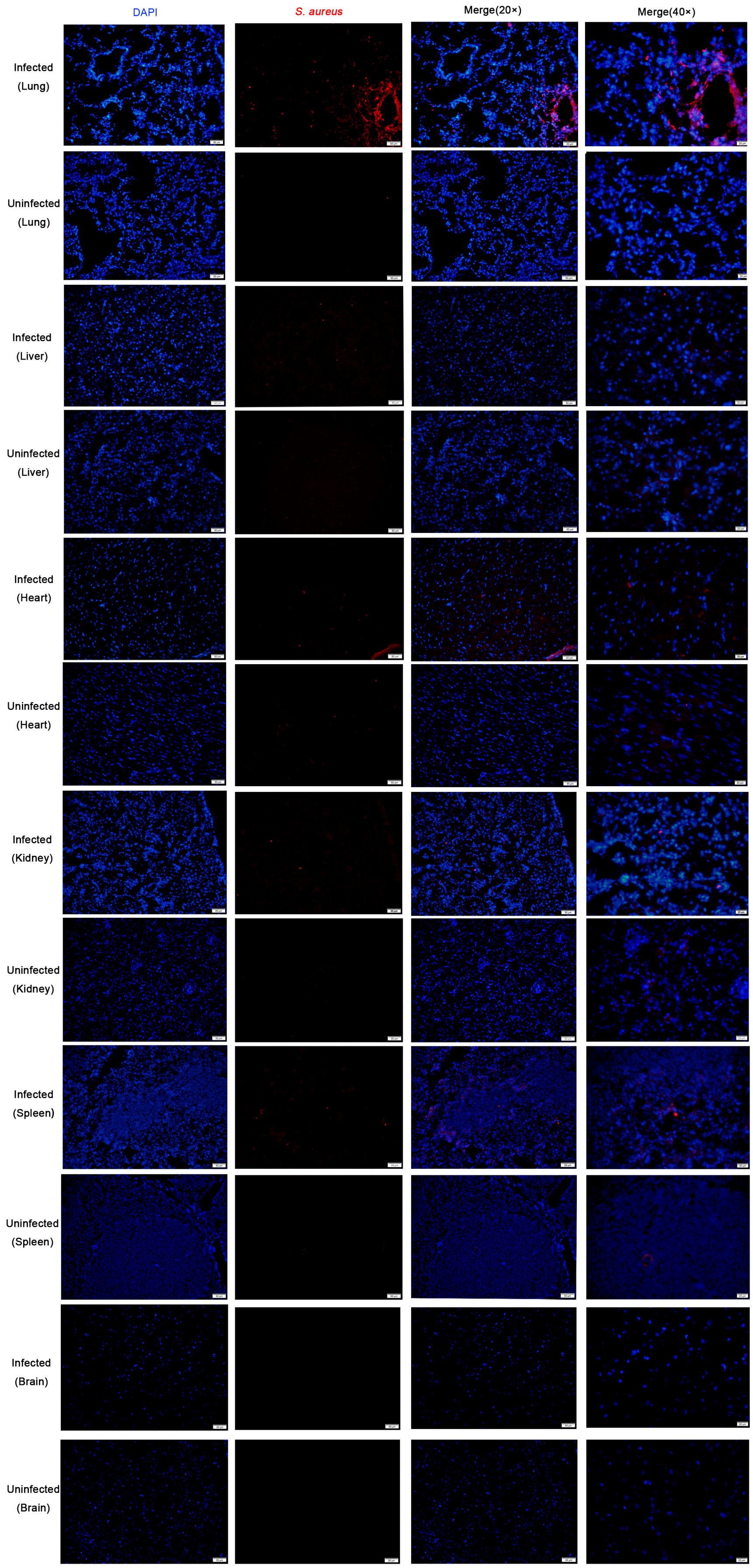
DAPI
S. aureus
Merge(20×)
Merge(40×)
Infected (Lung)
Uninfected (Lung)
Infected (Liver)
Uninfected (Liver)
Infected (Heart)
Uninfected (Heart)
Infected (Kidney)
Uninfected (Kidney)
Infected (Spleen)
Uninfected (Spleen)
Infected (Brain)
Uninfected (Brain)

Supplementary Figure S3 Distribution characteristics of bacteria in organs of mouse model. Sections were immunostained with primary antibodies specific to the SPA protein of *Staphylococcus aureus.* Bacteia and DAPI are in red and blue, respectively. Arrows indicate bacteria positive staining. Infected: MRSA T144 strain-infected group; Uninfected: blank control.

Supplementary Table S4 Strain number and Locatiou of 723 strains in phylogenetic tree.

Strain	Location	Strain	Location	Strain	Location
GCF_000009585.1	Netherlands	GCF_004785995.1	USA	GCF_026625365.1	China
GCF_000009645.1	Netherlands	GCF_005705815.1	Pakistan	GCF_026625385.1	China
GCF_000009665.1	Japan	GCF_005707215.1	Pakistan	GCF_026625405.1	China
GCF_000010445.1	Japan	GCF_005707335.1	Pakistan	GCF_026625445.1	China
GCF_000011265.1	Japan	GCF_005885975.1	USA	GCF_026625465.1	China
GCF_000011505.1	USA	GCF_005889995.1	USA	GCF_026625505.1	China
GCF_000012045.1	USA	GCF_005890015.1	USA	GCF_026625525.1	China
GCF_000013465.1	USA	GCF_005890035.1	USA	GCF_026625545.1	China
GCF_000016805.1	USA	GCF_005954685.1	USA	GCF_027920385.1	Australia
GCF_000017085.1	USA	GCF_006158985.1	China	GCF_027942255.1	China
GCF_000025145.1	Germany	GCF_006364775.1	USA	GCF_027942275.1	China
GCF_000027045.1	United Kingdom	GCF_007185035.1	Japan	GCF_028596045.1	USA
GCF_000144955.2	Australia	GCF_007410625.1	USA	GCF_029094145.1	USA
GCF_000145595.1	Australia	GCF_007556695.2	USA	GCF_029094305.1	USA
GCF_000159535.2	USA	GCF_007954445.1	USA	GCF_029167685.1	China
GCF_000204665.1	China	GCF_007954465.1	USA	GCF_029167705.1	China
GCF_000237125.2	China	GCF_008244705.1	Australia	GCF_029204095.1	China

GCF_000239235.1	Denmark	GCF_008244725.1	Australia	GCF_029204125.1	China
GCF_000284535.1	United Kingdom	GCF_008244745.1	Australia	GCF_029204145.1	China
GCF_000296595.1	Canada	GCF_008329865.1	USA	GCF_029204165.1	China
GCF_000382965.1	Switzerland	GCF_008329885.1	USA	GCF_029278505.1	China
GCF_000382985.1	Switzerland	GCF_008330045.1	China	GCF_029625375.1	China
GCF_000383005.1	Switzerland	GCF_008370815.1	USA	GCF_030014065.1	Belgium
GCF_000412775.1	USA	GCF_008619075.1	Japan	GCF_030014085.1	Belgium
GCF_000418345.1	Brazil	GCF_008619235.1	Japan	GCF_030015595.1	Belgium
GCF_000463055.1	USA	GCF_008619415.1	Japan	GCF_030168515.1	China
GCF_000470845.1	China	GCF_008619935.1	Japan	GCF_030252695.1	Germany
GCF_000470865.1	China	GCF_008620055.1	Japan	GCF_030252735.1	Germany
GCF_000485885.1	China	GCF_008693765.1	USA	GCF_030252755.1	Germany
GCF_000568455.1	USA	GCF_009176765.1	Japan	GCF_030262395.2	USA
GCF_000626615.2	USA	GCF_009176785.1	Japan	GCF_030262405.2	USA
GCF_000695215.1	Belgium	GCF_009176875.1	Japan	GCF_030262455.2	USA
GCF_000695875.1	Belgium	GCF_009684655.1	China	GCF_030342445.2	USA
GCF_000737615.1	China	GCF_009739675.1	Israel	GCF_030342475.2	USA
GCF_000746505.1	USA	GCF_009739695.1	Israel	GCF_030345195.1	Switzerland
GCF_000769575.1	Brazil	GCF_009739715.1	Israel	GCF_030345215.1	Switzerland
GCF_000815045.1	USA	GCF_009739735.1	Israel	GCF_030345235.1	Switzerland
GCF_000815085.1	USA	GCF_009739755.1	Israel	GCF_030382025.2	USA

GCF_000815125.1	USA	GCF_009739775.1	Israel	GCF_030408935.1	USA
GCF_000815165.1	USA	GCF_009739795.1	Israel	GCF_030408955.1	USA
GCF_000815205.1	USA	GCF_009739825.1	Israel	GCF_030408975.1	USA
GCF_000815245.1	USA	GCF_009739845.1	Israel	GCF_030408995.1	USA
GCF_000828035.1	Australia	GCF_009739865.1	Israel	GCF_030409015.1	USA
GCF_000967325.1	Switzerland	GCF_009739885.1	Israel	GCF_030409035.1	USA
GCF_000967345.1	Switzerland	GCF_009740205.1	Israel	GCF_030435875.1	South Korea
GCF_000967365.1	Switzerland	GCF_009857055.1	China	GCF_030533465.1	China
GCF_000967385.1	Switzerland	GCF_009912895.1	Germany	GCF_030533495.1	China
GCF_000967405.1	Switzerland	GCF_009912975.1	Germany	GCF_030533515.1	China
GCF_000969225.1	Brazil	GCF_009912995.1	Germany	GCF_030533555.1	China
GCF_001018835.2	USA	GCF_009913015.1	Germany	GCF_030533585.1	China
GCF_001019125.2	USA	GCF_009913095.1	Germany	GCF_030533605.1	China
GCF_001019205.2	USA	GCF_009913115.1	Germany	GCF_030533645.1	China
GCF_001019255.2	USA	GCF_009913135.1	Germany	GCF_030533665.1	China
GCF_001019305.2	USA	GCF_009913155.1	Germany	GCF_030533725.1	China
GCF_001019395.2	USA	GCF_009913175.1	Germany	GCF_030533745.1	China
GCF_001019435.2	USA	GCF_009913195.1	Germany	GCF_030533765.1	China
GCF_001019485.2	USA	GCF_009914455.1	China	GCF_030533785.1	China
GCF_001019495.2	USA	GCF_010669265.1	China	GCF_030533805.1	China
GCF_001019535.2	USA	GCF_011035105.1	Denmark	GCF_030533835.1	China

GCF_001019575.2	USA	GCF_013003945.1	Korea	GCF_030533855.1	China
GCF_001021875.1	USA	GCF_013008375.1	Germany	GCF_030533875.1	China
GCF_001021895.1	USA	GCF_013046865.1	China	GCF_030533895.1	China
GCF_001045795.2	USA	GCF_013046885.1	China	GCF_030533915.1	China
GCF_001045995.2	USA	GCF_013305905.1	USA	GCF_030533995.1	China
GCF_001046095.2	USA	GCF_013307005.2	USA	GCF_030534015.1	China
GCF_001183705.3	USA	GCF_013307025.1	USA	GCF_030534075.1	China
GCF_001183725.2	USA	GCF_013307065.1	USA	GCF_030534095.1	China
GCF_001296985.1	Netherlands	GCF_013389715.1	Belarus	GCF_030534135.1	China
GCF_001298325.2	China	GCF_013394425.1	Japan	GCF_030534235.1	China
GCF_001307235.1	Denmark	GCF_013415955.1	Brazil	GCF_030534255.1	China
GCF_001457495.1	United Kingdom	GCF_013467405.1	South Korea	GCF_030758795.1	USA
GCF_001465635.1	Netherlands	GCF_013836525.1	Denmark	GCF_030758815.1	USA
GCF_001465675.1	Netherlands	GCF_013836745.1	Denmark	GCF_030758835.1	USA
GCF_001465755.1	Netherlands	GCF_014696255.1	Canada	GCF_030758855.1	USA
GCF_001515665.1	Brazil	GCF_014696685.1	Canada	GCF_031190615.1	South Korea
GCF_001515685.1	Brazil	GCF_014696975.1	Canada	GCF_031326145.1	South Korea
GCF_001515705.1	Brazil	GCF_015219885.1	India	GCF_031826655.1	South Korea
GCF_001515745.1	Brazil	GCF_015219905.1	India	GCF_031826815.1	USA
GCF_001515765.1	Brazil	GCF_015220695.1	India	GCF_031826835.1	USA
GCF_001548415.1	Japan	GCF_015475575.1	USA	GCF_032808505.1	Italy

GCF_001549655.1	Japan	GCF_015767475.1	China	GCF_032809245.1	Italy
GCF_001549675.1	Japan	GCF_015767495.1	China	GCF_033802645.2	Thailand
GCF_001580495.1	South Korea	GCF_016065215.1	Germany	GCF_033808255.1	USA
GCF_001580515.1	USA	GCF_016496025.1	Germany	GCF_033952345.2	USA
GCF_001618305.1	Netherlands	GCF_016591995.1	Japan	GCF_033985535.2	USA
GCF_001640885.1	China	GCF_017088305.1	Australia	GCF_034105255.2	USA
GCF_001640905.1	Korea	GCF_017088385.1	Australia	GCF_034424945.2	USA
GCF_001640925.1	Korea	GCF_018135565.1	Switzerland	GCF_034424965.2	USA
GCF_001641025.1	Korea	GCF_018336515.1	China	GCF_034425095.2	USA
GCF_001641045.1	Korea	GCF_018336535.1	China	GCF_034425135.2	USA
GCF_001717645.1	USA	GCF_018409145.1	Japan	GCF_034427895.2	USA
GCF_001717685.1	USA	GCF_018987325.1	Japan	GCF_034554895.2	USA
GCF_001717705.1	USA	GCF_018987345.1	Japan	GCF_034554905.2	USA
GCF_001717725.1	USA	GCF_018987365.1	China	GCF_034661335.1	South Korea
GCF_001725965.1	USA	GCF_019134615.1	China	GCF_034809525.1	South Korea
GCF_001735655.2	USA	GCF_019550875.1	South Korea	GCF_034809545.1	Sweden
GCF_001880265.1	China	GCF_019550945.1	South Korea	GCF_036670045.1	USA
GCF_001887075.1	Canada	GCF_019551035.1	South Korea	GCF_036689715.2	USA
GCF_001956815.1	USA	GCF_019551095.1	South Korea	GCF_036697825.2	USA
GCF_001975005.1	USA	GCF_019551175.1	South Korea	GCF_036867125.2	USA
GCF_001975045.1	USA	GCF_019551235.1	South Korea	GCF_036867165.2	USA

GCF_0019861<br>35.1	China	GCF_019551<br>315.1	South Korea	GCF_036961<br>545.2	USA
GCF_0020005<br>65.1	Netherlands	GCF_019551<br>355.1	South Korea	GCF_037039<br>295.1	Germany
GCF_0020005<br>85.1	Netherlands	GCF_019551<br>375.1	South Korea	GCF_037039<br>325.1	Germany
GCF_0020006<br>05.1	Netherlands	GCF_019551<br>415.1	South Korea	GCF_037039<br>335.1	Germany
GCF_0020006<br>25.1	Netherlands	GCF_019603<br>375.1	South Korea	GCF_037045<br>985.2	USA
GCF_0020006<br>45.1	Netherlands	GCF_019653<br>615.1	Germany	GCF_037046<br>245.2	USA
GCF_0020006<br>65.1	Netherlands	GCF_019669<br>165.1	Japan	GCF_037046<br>505.2	USA
GCF_0020006<br>85.1	Netherlands	GCF_019669<br>245.1	Japan	GCF_037076<br>495.1	Japan
GCF_0020007<br>05.1	Suriname	GCF_019669<br>345.1	Japan	GCF_037076<br>505.1	Japan
GCF_0020007<br>25.1	Suriname	GCF_019738<br>975.1	USA	GCF_037076<br>515.1	Japan
GCF_0020007<br>45.1	Suriname	GCF_019915<br>585.1	South Korea	GCF_037083<br>885.2	USA
GCF_0020007<br>65.1	Suriname	GCF_020003<br>145.1	Netherlands	GCF_037095<br>035.2	USA
GCF_0020007<br>85.1	Suriname	GCF_020003<br>165.1	Netherlands	GCF_037095<br>045.2	USA
GCF_0020008<br>05.1	Suriname	GCF_020034<br>535.1	South Korea	GCF_037199<br>565.1	USA
GCF_0020008<br>25.1	Suriname	GCF_020042<br>725.1	China	GCF_037199<br>595.2	USA
GCF_0020008<br>45.1	Suriname	GCF_020097<br>755.1	China	GCF_037199<br>715.2	USA
GCF_0020008<br>65.1	Suriname	GCF_020150<br>915.1	China	GCF_037199<br>755.2	USA
GCF_0020251<br>25.1	Germany	GCF_020177<br>155.3	Malaysia	GCF_037217<br>515.1	USA
GCF_0020855<br>25.1	USA	GCF_020702<br>515.2	China	GCF_037217<br>645.1	USA
GCF_0020889<br>95.1	Canada	GCF_020702<br>545.2	China	GCF_037219<br>465.1	USA
GCF_0020890<br>55.1	China	GCF_020702<br>575.2	China	GCF_037414<br>495.1	USA

GCF_002097595.2	Canada	GCF_020702585.2	China	GCF_037482255.1	USA
GCF_002140115.1	South Korea	GCF_020702615.2	China	GCF_037482265.1	USA
GCF_002204555.1	USA	GCF_020881895.1	China	GCF_037938875.1	USA
GCF_002204575.1	USA	GCF_020887155.1	Japan	GCF_037948675.1	USA
GCF_002214665.1	South Korea	GCF_021172885.1	USA	GCF_037951045.1	USA
GCF_002355355.1	Japan	GCF_021172905.1	USA	GCF_037993065.1	New Zealand
GCF_002356675.1	Japan	GCF_021172925.1	USA	GCF_038024215.1	USA
GCF_002442975.1	China	GCF_021172945.1	USA	GCF_038024225.1	USA
GCF_002803885.1	Denmark	GCF_021249285.1	Ireland	GCF_038024695.1	Japan
GCF_002895385.1	China	GCF_021869805.1	USA	GCF_038024705.1	Japan
GCF_003029645.1	USA	GCF_022221525.1	China	GCF_038024715.1	Japan
GCF_003030065.1	USA	GCF_022221545.1	China	GCF_038024725.1	Japan
GCF_003031485.1	Brazil	GCF_022221565.1	China	GCF_038024735.1	Japan
GCF_003073395.1	Spain	GCF_022369595.1	USA	GCF_038024745.1	Japan
GCF_003073415.1	Spain	GCF_022404615.1	USA	GCF_038024755.1	Japan
GCF_003073435.1	Spain	GCF_022405145.1	USA	GCF_038024985.1	Japan
GCF_003073635.1	USA	GCF_022405355.1	USA	GCF_038025015.1	Japan
GCF_003073655.1	USA	GCF_022405895.1	USA	GCF_038025155.1	Japan
GCF_003073715.1	USA	GCF_022494545.1	China	GCF_038048995.1	USA
GCF_003073755.1	USA	GCF_022494565.1	China	GCF_038096205.1	USA
GCF_003073775.1	USA	GCF_022532145.1	China	GCF_038096225.1	USA

GCF_003111725.1	Australia	GCF_022682325.1	China	GCF_038096235.1	USA
GCF_003111745.1	Australia	GCF_022691325.1	USA	GCF_038096265.1	USA
GCF_003193665.1	USA	GCF_022691345.1	USA	GCF_038096285.1	USA
GCF_003193685.1	USA	GCF_022691365.1	USA	GCF_038096295.1	USA
GCF_003193705.1	USA	GCF_022691385.1	USA	GCF_038096305.1	USA
GCF_003193725.1	USA	GCF_022691405.1	USA	GCF_038096315.1	USA
GCF_003193745.1	USA	GCF_022691425.1	USA	GCF_038115185.1	USA
GCF_003193765.1	USA	GCF_022693165.1	USA	GCF_038129325.1	USA
GCF_003193785.1	USA	GCF_022693185.1	USA	GCF_038426605.1	China
GCF_003193885.1	USA	GCF_022693245.1	USA	GCF_038427395.1	China
GCF_003193965.1	USA	GCF_022693265.1	USA	GCF_038428225.1	China
GCF_003194005.1	Brazil	GCF_022693285.1	USA	GCF_038429035.1	China
GCF_003194025.1	Brazil	GCF_022693305.1	USA	GCF_038429865.1	China
GCF_003194405.1	Brazil	GCF_022693325.1	USA	GCF_038429905.1	China
GCF_003203595.1	USA	GCF_022699245.1	Australia	GCF_038429955.1	China
GCF_003203615.1	USA	GCF_022699265.1	Australia	GCF_038430005.1	China
GCF_003203635.1	USA	GCF_022699285.1	Australia	GCF_038430115.1	China
GCF_003203675.1	USA	GCF_022809795.1	China	GCF_038786585.1	Germany
GCF_003264775.1	Canada	GCF_022832755.1	China	GCF_038788575.1	Germany
GCF_003264815.1	Canada	GCF_022832835.1	China	GCF_038789165.1	Germany
GCF_003288395.1	USA	GCF_022870005.1	USA	GCF_038789195.1	Germany

GCF_003330905.1	USA	GCF_022870625.1	USA	GCF_039830175.1	USA
GCF_003342735.1	South Korea	GCF_023170025.1	Japan	GCF_040047685.1	South Korea
GCF_003351925.1	South Korea	GCF_023170045.1	Japan	GCF_040315025.1	Czech
GCF_003354565.1	USA	GCF_023223475.1	Germany	GCF_040315155.1	Czech
GCF_003354585.1	USA	GCF_023373745.1	China	GCF_040361455.1	China
GCF_003354665.1	USA	GCF_024172245.1	USA	GCF_040361795.1	China
GCF_003354705.1	USA	GCF_024205325.1	China	GCF_040362785.1	China
GCF_003354765.1	USA	GCF_024363305.1	Japan	GCF_040365755.1	China
GCF_003354945.1	USA	GCF_024363325.1	Japan	GCF_040931685.1	Japan
GCF_003354965.1	Germany	GCF_024363345.1	Japan	GCF_040931695.1	Japan
GCF_003354985.1	USA	GCF_024363365.1	Japan	GCF_040931705.1	Japan
GCF_003394105.1	Australia	GCF_024363385.1	Japan	GCF_040931715.1	Japan
GCF_003431365.1	USA	GCF_024363445.1	Japan	GCF_040931725.1	Japan
GCF_003432345.1	China	GCF_024363465.1	Japan	GCF_040931735.1	Japan
GCF_003432365.1	China	GCF_024363485.1	Japan	GCF_040931745.1	Japan
GCF_003573835.1	Japan	GCF_024363505.1	Japan	GCF_040931755.1	Japan
GCF_003595365.1	Suriname	GCF_024925485.1	Czech	GCF_040931765.1	Japan
GCF_003595385.1	Suriname	GCF_025152705.1	USA	GCF_040931775.1	Japan
GCF_003595405.1	Suriname	GCF_025232045.1	Japan	GCF_040931785.1	Japan
GCF_003595425.1	Suriname	GCF_025259685.1	USA	GCF_040931795.1	Japan
GCF_003595445.1	Suriname	GCF_025398055.1	China	GCF_040931805.1	Japan

GCF_003595465.1	Suriname	GCF_026546985.1	Netherlands	GCF_040931815.1	Japan
GCF_003595485.1	Suriname	GCF_026547005.1	Netherlands	GCF_040931825.1	Japan
GCF_003595505.1	Suriname	GCF_026547035.1	Netherlands	GCF_040931835.1	Japan
GCF_003827915.1	USA	GCF_026547055.1	Netherlands	GCF_040931845.1	Japan
GCF_003945425.1	Japan	GCF_026547075.1	Netherlands	GCF_040931865.1	Japan
GCF_003991015.1	USA	GCF_026547095.1	Netherlands	GCF_040931875.1	Japan
GCF_004026165.1	South Korea	GCF_026547145.1	Netherlands	GCF_040931885.1	Japan
GCF_004026185.1	South Korea	GCF_026547185.1	Netherlands	GCF_040931895.1	Japan
GCF_004118995.1	USA	GCF_026625205.1	China	GCF_040931905.1	Japan
GCF_004136235.1	China	GCF_026625225.1	China	GCF_040931915.1	Japan
GCF_004136255.1	China	GCF_026625245.1	China	GCF_040931925.1	Japan
GCF_004136655.1	China	GCF_026625265.1	China	GCF_040931935.1	Japan
GCF_004153365.1	India	GCF_026625285.1	China	GCF_040931945.1	Japan
GCF_004208595.1	USA	GCF_026625305.1	China	GCF_040931955.1	Japan
GCF_004614295.1	Canada	GCF_026625325.1	China	GCF_040931965.1	Japan
GCF_004614315.1	Canada	GCF_026625345.1	China	GCF_040931975.1	Japan
GCF_040931985.1	Japan	GCF_041902405.1	Switzerland	GCF_045346835.1	Japan
GCF_041200075.1	USA	GCF_041902415.1	Switzerland	GCF_045346865.1	Japan
GCF_041464255.1	USA	GCF_041902425.1	Switzerland	GCF_045346955.1	Japan
GCF_041464265.1	USA	GCF_041927245.1	France	GCF_045347025.1	Japan
GCF_041464275.1	USA	GCF_042078115.1	South Korea	GCF_045347075.1	Japan

GCF_041517615.1	USA	GCF_042753635.1	South Korea	GCF_045347165.1	Japan
GCF_041518565.1	USA	GCF_044496705.1	Thailand	GCF_045347195.1	Japan
GCF_041519015.1	USA	GCF_044497995.1	China	GCF_045347285.1	Japan
GCF_041519025.1	USA	GCF_044789125.1	Russia	GCF_045347315.1	Japan
GCF_041519035.1	USA	GCF_045009735.1	Thailand	GCF_045347385.1	Japan
GCF_041519385.1	USA	GCF_045009745.1	Thailand	GCF_045347455.1	Japan
GCF_041519415.1	USA	GCF_045010355.1	Thailand	GCF_045347525.1	Japan
GCF_041531605.1	USA	GCF_045010375.1	Thailand	GCF_045347575.1	Japan
GCF_041531615.1	USA	GCF_045011635.1	Bulgaria	GCF_045348335.1	USA
GCF_041531645.1	USA	GCF_045278255.1	China	GCF_045348345.1	USA
GCF_041531655.1	USA	GCF_045345275.1	Australia	GCF_045348355.1	USA
GCF_041531685.1	USA	GCF_045346175.1	Japan	GCF_900017775.1	Australia
GCF_041697955.1	USA	GCF_045346275.1	Japan	GCF_900129335.1	Germany
GCF_041697965.1	USA	GCF_045346335.1	Japan	GCF_900324205.1	Germany
GCF_041697975.1	USA	GCF_045346405.1	Japan	GCF_900324215.1	Germany
GCF_041697985.1	USA	GCF_045346515.1	Japan	GCF_900324225.1	Germany
GCF_041733125.1	USA	GCF_045346525.1	Japan	GCF_900324235.1	Germany
GCF_041734105.1	USA	GCF_045346615.1	Japan	GCF_900324245.1	Germany
GCF_041734765.1	USA	GCF_045346645.1	Japan	GCF_900324255.1	Germany
GCF_041874635.1	USA	GCF_045346715.1	Japan	GCF_900324265.1	Germany
GCF_041879455.1	USA	GCF_045346765.1	Japan	GCF_964057045.1	Netherlands

GCF_900324285.1	Germany	GCF_900607275.1	Australia	GCF_964057085.1	Netherlands
GCF_900324295.1	Germany	GCF_900607285.1	Australia	GCF_964057115.1	Netherlands
GCF_900324305.1	Germany	GCF_900607295.1	Australia	GCF_964057125.1	Netherlands
GCF_900324315.1	Germany	GCF_900607305.1	Australia	GCF_964057135.1	Netherlands
GCF_900324325.1	Germany	GCF_900620235.1	Australia	GCF_964057145.1	Netherlands
GCF_900324335.1	Germany	GCF_900620245.1	Australia	GCF_964057155.1	Netherlands
GCF_900324345.1	Germany	GCF_900620255.1	Australia	GCF_964057165.1	Netherlands
GCF_900324355.1	Germany	GCF_900635245.1	United Kingdom	MRSA144	China
GCF_900324365.1	Germany	GCF_900635265.1	United Kingdom	GCF_900478245.1	United Kingdom
GCF_900324385.1	Germany	GCF_900635905.1	United Kingdom	GCF_900607245.1	Australia
GCF_900324405.1	Germany	GCF_903932595.1	Argentina	GCF_900607255.1	Australia
GCF_900324415.1	Germany	GCF_903932605.1	Argentina	GCF_900607265.1	Australia
GCF_900474525.1	United Kingdom	GCF_947034825.1	France	GCF_900474735.1	United Kingdom
GCF_900474575.1	United Kingdom	GCF_900474725.1	United Kingdom		
